# Challenges to Establish Effective Public-Private Partnerships to Address Malnutrition in All Its Forms

**DOI:** 10.34172/ijhpm.2020.262

**Published:** 2021-01-16

**Authors:** Jessica Fanzo, Yusra Ribhi Shawar, Tara Shyam, Shreya Das, Jeremy Shiffman

**Affiliations:** ^1^The Nitze School of Advanced International Studies, Johns Hopkins University, Baltimore, MD, USA.; ^2^The Bloomberg School of Public Health, Johns Hopkins University, Baltimore, MD, USA.; ^3^The Berman Institute of Bioethics, Johns Hopkins University, Baltimore, MD, USA.; ^4^Independent Consultant, Singapore, Singapore.; ^5^Independent Consultant, New Delhi, India.

**Keywords:** Public-Private Partnerships, Food Systems, Nutrition, Accountability, Trust, Transparency

## Abstract

**Background:** Every country is affected by some form of malnutrition. Some governments and nutrition experts look to public-private partnerships (PPPs) to address the burden of malnutrition. However, nutrition-related PPPs face opposition, are difficult to form, and there is limited evidence of their effectiveness.

**Methods:** We conducted a literature review and 30 semi-structured interviews with individuals involved in or researching nutrition-related PPPs to identify the factors that shape their creation and effectiveness in food systems.

**Results:** Several factors make it difficult to establish nutrition-related PPPs in food systems: a lack of understanding of the causal pathways behind many nutrition problems; a weak architecture for the global governance of nutrition; power imbalances between public and private sector nutrition actors; and disagreements in the nutrition community on the advisability of engaging the private sector. These complexities in turn make it difficult for PPPs to be effective once established due to goal ambiguity and misalignment, resource imbalances, and weak accountability.

**Conclusion:** If effective nutrition-related PPPs are to be established, private sector conflicts of interest must be addressed, trust deficits between private and public sector actors must be surmounted, and evidence must be assessed as to whether PPPs can achieve more for public health nutrition than private and public sector actors working separately.

## Introduction

Every country is affected by malnutrition in some form, be it undernutrition, micronutrient deficiencies, or overweight and obesity, with some countries struggling with multiple forms.^1,2^ One of the most impactful solutions to address this challenge is to improve the nutrient-density of diets and dietary patterns for populations to reduce all forms of malnutrition.^3-7^ To do this, actions need to be taken through interconnected food systems. But diets are changing along with dynamic development, urbanization, and shifting demographics.^8^ Sub-optimal diets are now one of the top risk factors globally for deaths and disability-adjusted life-years lost due to non-communicable diseases (NCDs), surpassing tobacco smoking and high blood pressure.^9^ To realign food systems and to respond to dietary transitions, it is important to establish accountability mechanisms and incentives that aim to improve the affordability and accessibility of foods and beverages that support healthy diets and address all forms of malnutrition.^10-14^

Both the private and public sectors play significant roles in shaping diets through the food supply and food environments.^15,16^ The majority of food consumed by the world’s population involves a “broad range of commercial enterprises” – from large, multi-nationals to small- and medium-scale enterprises (SMEs) involved in food transformation including agribusiness, food product processing, reformulation and packaging, meal manufacturers, and the advertising and marketing industry.^17^ These enterprises are made up of a diverse set of actors including food and beverage manufacturers, retailers, food service providers, industry trade associations, wholesalers, distributors, importers and exporters.^15^ They range from informal, less structured small enterprises to well-organized, large-scale trans-national companies. There are also actors that are not necessarily directly involved in the food system and its activities but their products and services influence food system change, such as the mobile phone industry and communications agencies.

The private sector also has significant power across food systems, with involvement in almost all aspects of the production, processing, distribution, marketing and sale of food that consumers eat every day.^18-20^The private sector has the capacity for market penetration far beyond that of the public health sector.^21^ Whilst the private sector has made innovations to promote healthy lifestyles^22^ or improve the nutrition of their portfolio of products (ie, fortification), a significant proportion of food products do not contribute to healthy lifestyles and diets in general, and many food and beverage companies do not fully align with consumer health.^23,24^

Many of the actors working in and governing food systems are calling for the private sector, in all its shapes, forms and sizes, along with its supporting ecosystem, to become increasingly aligned with public nutrition and health policy. Some look to public-private partnerships (PPPs) as a potential vehicle for realizing public health goals — in this case public health nutrition — while stimulating private investment and additional resources to foster the development of food systems that provide healthier foods.^25-30^ PPPs are alliances designed to achieve common goals benefiting societies and all partners^31-33^ but can entail a mixture of interactions involving a range of different activities, processes and structures.^34^

There is a range of partnerships in nutrition depending on the end goal. They can be research driven, commercially focused, or designed to promote public health initiatives. Some partnerships involve a minimal level of commitment from each partner, whereas in other situations the partnership involves greater commitment with pooled resources, shared decision-making and obligations.^35^ Between these two extremes lie a wide range of levels of commitment between partners.^33^ There are also public private engagements which is a broader area capturing all modalities of engagement between the public and private sectors from informal collaborations to more formalized partnerships.^36^

The World Health Organization (WHO) defines PPPs as “a collaboration between public-and private-sector actors within diverse arrangements that vary according to participants, legal status, governance, management, policy setting, contributions and operational roles to achieve specific outcomes.”^37^ In the field of nutrition, there is no single definition of a PPP, no consensus on how they should be defined and inadequate documentary evidence of the efficacy of such partnerships.^34,38-40^

Ideally, PPPs should be designed first and foremost to benefit public health goals, in this case, public health nutrition, while stimulating private investment and additional resources to foster development. Discussion around the effectiveness of PPPs is polarized within the food systems community, and in particular, in the field of nutrition.^16,33,41-42^ On the one hand, some argue that PPPs offer the potential to improve diet and nutrition outcomes by harnessing resources, reach, relationships and knowledge from both government and private sector actors.^1,42^ Others have reservations about engaging with food and beverage industries, deeming them as major contributors to the malnutrition burden.^1,43-46^ “The question, then, is what is the best way to work with industry actors, whose products contribute to chronic diseases, and whose practices undermine policy responses to NCDs, without jeopardizing public welfare.”^46^

 In this paper, we investigated the challenges of establishing PPPs in food systems that are focused on improving diets and nutrition, and why they struggle to succeed. We identified the factors shaping the formation and development of PPPs, highlighting challenges to their emergence as well as obstacles that facilitate their growth and effectiveness in contributing to public health and nutrition policy. This paper focuses only on PPPs that take place in food systems with an aim to improve diets and nutrition. Although there are many other types of PPPs that are geared towards improving nutrition, and many other types of PPPs within food systems that shape other outcomes (eg, economic growth, agriculture productivity and environmental sustainability), taking a broader approach is beyond the remit of this paper.

## Key Messages

**Implications for policy makers**

- Policy-makers should decide if a partnership with private sector is warranted and if so, that there is sufficient causal pathways to impact, strong governance mechanisms, and no conflicts of interest between actors of the partnership.
- If policy-makers are to partner with private sector, the architecture of the engagement needs to be transparent, open, and inclusive with a main goal to improve public health nutrition.
- Putting in place robust accountability mechanisms within established public-private partnerships (PPPs) will be critical for governments so that they can steward, monitor and incentivize private sector actors to work towards promoting healthy diets and nutrition within food systems.

**Implications for public**

If governments are to engage with private sector food system actors, they need to exert their power and ensure that they are protecting citizens and promoting public health through those engagements. They cannot be passive bystanders. Our research found that nutrition-related public-private partnerships (PPPs) in food systems often fail because of a lack of governance, power imbalances and mistrust. Because of these challenges, consumers are often weary of food and beverage industry’s motives towards improvements in public health. Outside of PPPs, consumers look to governments to keep these industry players in check by developing evidence-based food-based dietary guidelines, supporting fiscal instruments such as taxes on soda and unhealthy junk food, and regulating advertising junk food to children. We hope this research will eventually change policies so that PPPs can be more effective in ensuring that food systems work for citizens by prioritizing public health nutrition before any other motives.

## Methods

###  Data Collection

We collected information on PPPs by searching Google Scholar and PubMed since 2000. Eighteen search terms and their combinations were used and are shown in Table 1. We restricted the literature review to articles in English that were broadly associated with PPPs or private sector involvement in the nutrition field. We excluded articles that did not discuss PPP emergence and/or effectiveness in the nutrition field. The search resulted in 113 peer review and grey literature articles which were then divided into the following categories: critiques, reviews or analyses of nutrition-focused PPPs; private sector involvement in nutrition research; case studies of nutrition-focused PPPs; and other PPPs with a focus on public health or agriculture.

**Table 1:** Search Terms for Literature Review

**Combined Searches With “Nutrition”**	**Combined Searches With “PPPs”**
Public-private partnerships	Diets
Industry	Food systems
Business	Food sector
Impact assessment PPPs	Nutrition
Collaborative governance	Healthy food
Commercial sector	Obesity
Market-based approach	Undernutrition
	Malnutrition
	Nutrition AND health outcomes
	Food safety

Abbreviation: PPPs, public-private partnerships.

We also conducted 30 semi-structured interviews by phone or Skype, between August 2018 and February 2019, with those who had researched PPPs, those directly involved with PPPs, and those with extensive knowledge of private or public sector involvement in nutrition. Using a purposive rather than sampling selection strategy, we selected individuals through the literature review and by asking those interviewed whom they considered to be most centrally involved in nutrition-focused PPPs, in academia and the public and private sectors. Key informants were from high-, middle- and low-income countries, representing various organizational affiliations with expertise in food systems and nutrition as shown in Table 2. One limitation of this sampling strategy is that it may be prone to selection bias. However, three of the authors of this study are outsiders to the nutrition field, and a purposeful effort was made to include a balanced reflection of perspectives from those engaged in both private and public sectors, as well as those involved in various subjects in the nutrition field. Given the lack of representation from those that come to nutrition from a position of engagement with undernutrition and micronutrient deficiencies as well as those from low- and middle-income settings, it would be valuable for future studies concerned with PPPs in nutrition to interview these actors as a means of further interrogating our findings. We continued interviewing until we reached theoretical saturation^47^ — the point at which all major themes have been identified and additional interviews are unlikely to reveal new information.

**Table 2:** Interviewee Affiliations

**Organization Type**	**Affiliation**
Academic/Research	City University of New York
	Cornell University
	Institute of Medicine
	International Food Policy Research Institute
	New York University
	Pennsylvania State University
	SR^a^ Strategy
	University of Guelph
	Virginia Tech
Private sector	BASF
	Compass Group Canada
	Emerging Ag Inc.
	Partnering with Purpose
	PepsiCo
	Royal DSM
	Unilever
	WBCSD
Public sector (including nonprofits)	1000 Days
	BRAC
	IDRC
	IFIC Foundation
	MicroNutrient Initiative
	PATH
	Save the Children USA
Combined private and public sector alliance	MotherFood International
	Sight and Life
	GAIN
	SUN Business Network
Intergovernmental organizations	UNICEF
	WHO

Abbreviations: BASF, Badische Anilin und Soda Fabrik; Royal DSM, Dutch State Mines; WBCSD, World Business Council for Sustainable Development; BRAC, Bangladesh Rural Advancement Committee (now Building Resources Across Communities); IDRC, International Development Research Centre; IFIC, International Food Information Council; PATH, Program for Appropriate Technology in Health GAIN, Global Alliance for Improved Nutrition; SUN, Scaling Up Nutrition; UNICEF, United Nations Children’s Fund; WHO, World Health Organization. ^a^SR is the name of an interviewee.

 The interviews lasted approximately one hour on average and were recorded and transcribed with permission from the key informants. We obtained informed consent from all research participants. All interview transcripts and notes were de-identified and secured in password-protected documents to ensure respondent confidentiality. Drawing on the findings from the literature review, questions were focused on successes and challenges of establishing high quality and impactful PPPs. We tailored questions to each interviewee’s expertise.

###  Data Analysis

To analyze factors shaping PPP establishment and effectiveness, we conducted a thematic analysis in Microsoft Word, drawing on information from key informant interviews and collected literature. Grounded in governance, policy and PPP-specific scholarship, ^48,49^ we initially coded the collected data under the broad categories of challenges and opportunities/strengths with sub-codes: issue characteristics (the nature of the issue), policy environment (the external policy and market context that PPPs operate in), PPP governance and management (the internal structure and operation of the PPP), and the actors involved (the nature and dynamics of the involved actors). These sub-codes were highlighted as being key general factors in determining network, organizational, and/or PPP establishment and effectiveness in the conceptual and theoretical scholarship. The sub-codes evolved as more empirical data — from the key informant interviews and literature — was collected. For example, causal ambiguity became a key sub-code under issue characteristics; goal alignment, resource acquisition, accountability, and power imbalance became prominent sub-codes under PPP governance and management; and mistrust and fragmentation became key sub-codes under actors involved. To minimize bias and validate the accuracy of the findings, three of the authors coded the data simultaneously and the data sources were triangulated, always corroborating information from interviews with written sources. In reporting the interview data, each key informant was assigned a number.

## Results

 A review of evidence from the literature and the interviews revealed four primary factors that make it difficult to establish nutrition-related PPPs. These factors in turn shape the quality and efficacy of nutrition-related PPPs once established: the research reveals that PPPs commonly face challenges in creating alignment and clarity of goals, acquiring and balancing resources and establishing robust accountability mechanisms.

###  Factors That Pose Challenges to Nutrition PPP Establishment

 Four sets of factors make it difficult to establish nutrition-related PPPs. The first concerns the complexity of nutrition’s causal pathways. The second relates to the global governance of nutrition. The third pertains to power imbalances between public and private sector actors in nutrition. The fourth concerns mistrust that pervades the nutrition space surrounding engaging the private sector. The prominence of each factor depends on the nutrition issue being addressed.

####  Nutrition’s Complex Causal Pathways

Nutrition’s causal pathways are complex. There are multiple contributing factors to undernutrition in the form of stunting and wasting, micronutrient deficiencies, overweight and obesity, and diet-related NCDs.^2^ Each of these manifestations is biologically multifarious and complex, with a range of contributing factors and outcomes on health and well-being.^50-53^ There are also gaps in knowledge on underlying and immediate undernutrition and obesity causes and determinants,^54-56^ as well as the varying impacts of interventions at different stages of the life cycle.^54,55^ For example, there are still gaps in evidence on how to effectively and sustainably prevent the onset of obesity through food-based solutions.^57-60^ Furthermore, there is a lack of data with robust metrics on what people consume, the cost of diets and food environments in low- and middle-income contexts.^61-63^ This makes it difficult to establish PPPs, given uncertainties about what to focus on, the role of each actor involved and the implementation of critical nutrition interventions such as shaping consumer behaviors and designing the choice architecture of food environments (I9, I12).^64^ Two interviewees (I9, I12) expressed similar concerns stemming from the literature that a lack of nuanced understanding of the causal pathways, the complexity of these pathways and the potential to do harm without a clear understanding of them, heighten reservations to act and partner. One respondent compared the difficulties of establishing causality and goals in nutrition with climate change:

*“In the case of climate change, you can [estimate] the aggregate impact of automobile emissions or coal plant emissions and the goal becomes reducing those emissions. But the ability to demonstrate the impact of that on the temperature of the oceans and halting the receding of glaciers is far down the road, right? So there’s an analogy there in nutrition of can we agree on some approximate endpoints or is the only relevant nutrition goal going to be the more ultimate, biological endpoints that we tend to care about?” *(I12).

There is no silver bullet to address malnutrition in all its forms given nutrition’s multi-temporal, multi-faceted, multi-sectoral, multi-disciplinary nature.^65,66^ This makes it difficult to measure impact and incentivize actors to come together to work on changing nutrition outcomes — important precursors for establishing a PPP.^17,40,67-69^ Policy-makers and implementers can be left in a void, without guidance to determine whom to partner with and how.^70^ One respondent noted this difficulty:

*“Structurally, our field is very multidimensional and complex. This is in contrast to PPPs in public works. The outcomes and what needs to be done is very clear in infrastructure PPPs, but indicators in nutrition [are] not very clear” *(I9).

However, tackling micronutrient deficiencies is an area of nutrition that is more tractable and has therefore seen clearer evidence of impact with isolated interventions, such as high-dose vitamin A supplementation programs implemented in many countries given to children under the age of five years.^71^ Because it is a supplement, governments, the United Nations (UN) and non-governmental organizations partner with vitamin A capsule manufacturers. Another example is ready-to-use therapeutic foods used to treat severe cases of acute malnutrition. Agencies such as the United Nations Children’s Fund (UNICEF) must engage the private sector to ensure quality control and consistent productivity of the product.^72,73^ As a result, the availability of literature on this type of intervention suggests PPPs lend themselves to micronutrient fortification, biofortification and supplementation initiatives, although independent evaluations that dissect how those PPPs functioned and what made them successful are few.^38^

There is also insufficient evidence and data that nutrition-related PPPs have made an impact on improving diets through the food systems lens.^34,74-77^ Most PPPs are still in early or pilot phases that do not allow for proper evaluation, and many exist as pilots with less certainty for scale-up to serve consumers that need interventions or provisions the most.^78^ In our own search, we found many examples of PPPs, but very few were examined by an external third party for their effectiveness or impact. There has been minimal evaluation of what has made PPPs effective and what has hindered their progress^79^ (I1, I5, I6, I12).

####  Dysfunctional Global Governance of Nutrition and its Actors

The second challenge concerns the actors in nutrition and the field’s governance structures. Levine and Kuczynski have argued that the nutrition field has a “dysfunctional international architecture,” is underfunded and has lackluster leadership.^65,80,81^ Observers describe the field as a loose collection of entities without unified vision.^65,80,81^ This paper was written over ten years ago however, and the field of nutrition has changed. Following the publication of that paper, the Scaling Up Nutrition movement was formed, and several *Lancet* series and Commissions on undernutrition, obesity and food systems have been published since, creating more understanding and evidence of what needs to be done to improve nutrition outcomes.

 Yet still, individuals working in specialized areas such as humanitarian relief, obesity and micronutrient deficiencies are often isolated from one another and other sectors. This fragmentation fuels duplication of efforts and competition for resources, rather than collaboration and partnership. Many of these communities, made up of numerous diverse players, have developed their own distinct ideologies or different world views, including on how to engage with private sector. This division also puts excessive pressure on governments to determine where and how they should act, and with whom they should align.

*“I see an inability to come together on a coherent set of demands within the nutrition community as a function of some people having stronger ideological opposition to the industry and PPPs than others” *(I12).

The issue of trust between some actors in the nutrition community and the private sector plays out in multiple and diverse ways when addressing malnutrition, some of that due to the different approaches taken when dealing with one type of burden.^82^ First, in the undernutrition space overall, programmatic and funding allocation decisions are framed as a stark choice between a preventative approach (also called a nutrition-sensitive approach), which addresses the underlying causes of malnutrition, and a treatment approach (also called a nutrition-specific approach), which addresses the immediate causes of undernutrition.^83^

 Second, there are divisions between those that work on humanitarian or emergency nutrition issues in shorter time scales versus those who work on longer-term nutrition development challenges. The rapid response needed in humanitarian work does not afford that community the time to reflect on broader ideologies with respect to working with the private sector (beyond standard institutional due diligence). Food assistance inherently needs private sector partnership to swiftly deliver food (and sometimes specialized foods in non-spoilage packaging) to hard-to-reach places through sophisticated logistical operations, and to prepare and provide supplementary food products, such as ready-to-use therapeutic foods to treat acute severe undernutrition.

Third, another fault line exists in the nutrition community between those who work on undernutrition and those who work on overweight, obesity and diet-related NCD agendas. Much of the obesity community has been hesitant to engage with private sector due to the latter’s production of energy dense, nutrient-poor, unhealthy food products that contribute to the burden of malnutrition and their design of food environments that tend to be coercive such as in the sales and advertising of junk food to children or inexpensive highly processed, unhealthy foods (also known as junk food that is high in added sugars, sodium and unhealthy fats).^42,84-88^

*“There is the challenge of speaking a common language and having trust in one another, and that is related, again, to knowing people on the personal level. How do you build confidence in one another if you’ve never worked together?” *(I18).

These differing world views and divergent interests create different relationships with the private sector, with varying degrees of trust and suspicion, and deep ideological fissures. These decisions are highly debated in the nutrition community, particularly in fora such as the Scaling Up Nutrition Movement and the United Nations Committee on World Food Security. Unresolved, these differences split practitioners into groups that use different programmatic models to address malnutrition. In addition, addressing these different forms of malnutrition presents unique opportunities, risks and histories of PPP arrangements. One example of success is the long-standing fortification programs in low- and middle-income countries which have engaged governments with the private sector in fortifying staple grains to improve micronutrient deficiencies.^89-92^ As a result, some public sector actors are more willing than others, or indeed consider it absolutely essential, to work with private sector. These decisions come down to individual researchers or organizations. This fragmentation is exacerbated by disagreements among nutrition actors on the advisability of working with the private sector, an issue discussed below.^40^

####  Power Imbalance in Nutrition That Favors the Private Sector

The third challenge relates to power imbalances that favor the private sector. Public institutions and non-profit organizations are often too weak to provide a counterbalance to private sector influence, creating disincentives for partnering with the private sector in the form of PPPs (I10, I14, I15).^93-97^ Private sector actors are involved in almost all aspects of the production, processing, distribution, marketing and sale of food, dominating many of these functions.^97^ Moreover, some governments find regulating the nutrition and food space challenging due to the significant power of the private sector.^98^ One example where government has used regulation is through government-led taxes on certain food and beverage commodities deemed to be unhealthy, such as soda. Taxes have worked in some places, such as Mexico,^98^ however in other places, such as New York City’s soda tax and Denmark’s saturated fat tax, regulation failed to get off the ground, in part due to private sector lobbying and messaging to the public.^99,100^ Larger, more established private sector actors, particularly those which have undergone consolidation and have significant shares of key markets, use their power to override government voice to set agendas.^43^Respondents speak to these power imbalances:

*“Food and beverage industry has incredible power. Not only economically, but with political power. So can you have dinner with the devil? Well, you need to really have your tools to defend from what they are going to do to you. So, probably, there’s a perception that we don’t have those tools” *(I14).

*“Big Food corporations have used nutritional positioning to bolster their power and influence in the sector. Through lobbying and participation in nutritionally focused public–private partnerships, they have directly sought to influence policy and governance. Through market dominance in the nutritionally enhanced food sector, and participation in nutrition-focused rule-setting activities in agri-food supply chains, they have gained power to influence policy agendas.”* ^47^

The public sector’s lack of influence can also be tied to market forces.^101^ One respondent below noted that even if governments were to gain power in this domain and create meaningful food-based dietary guidelines and public procurement programs,^14,102,103^ fiscal instruments such as taxes on soda and unhealthy junk food, or regulations on advertising junk food to children, market forces and consumer demands are still important drivers of change.^104^ Respondents (I2, I5, I8, I9, I15) noted these market forces of consumer demand, saying:

*“I think when we talk about PPPs in the context of nutrition, the first step is to acknowledge that, in fact, public players have very little influence on people’s diets and their behaviors” *(I10).

####  Mistrust of the Private Sector

The fourth challenge is that many public sector actors and researchers mistrust the private sector due to a long history of wrongdoings towards public health goals.^85,105-107^ One example is the consistent violation of the World Health Assembly adopted International Code of Marketing Breastmilk Substitutes (I6, 9, I12, I14, I16, I18). The Code is meant to protect exclusive breastfeeding of infants younger than six months, and to position it as a complement to other foods for older infants. It is intended to protect mothers, health workers and the health systems in which they work from commercial promotion of breastmilk substitutes that undermine breastfeeding. A Save the Children report found many examples of continued violations of the established Code by some manufacturers of breast-milk substitutes (I2, I6, I9, I12).^108^ If public sector organizations or individuals (both development practitioners or researchers) do get involved with Code violators, they are often shamed. Interviewees expressed that certain private sector organizations are perceived to be a “no go” for engaging with the public sector because of past behavior with breaking the Code:

*“The public nutrition sector doesn’t want to work with any of the multinationals that produce infant formula. So Danone, Nestle, and most of the dairy-related industries are not at the table or not accepted to work together” *(I18).

Another transgression is the rampant availability and aggressive advertising of cheap junk food and sugar sweetened beverages to children (I12, I14, I16, I18).^29,101,104,109^ One study found that junk food advertising by the private sector is nearly 30 times what governments spend on healthy eating in the United Kingdom.^110^ In the United States, children ages 2 to 11 see an average of 10 food advertisements a day with most of those marketing unhealthy foods.^111^ Respondents note reservations about establishing PPPs given the behavior of the large, multi- and trans-national companies in the food environment space:

*“[There is] nervousness about large food companies today. [There is] clear evidence of the effect of poor diets on non-communicable diseases and for a long time, the resistance of the food companies to acknowledge the role they are playing. To a large extent, they still try to work around that by placing the blame on individual behavior and lifestyles and choices rather than the environment that they are helping to create, not only the food environment, but the information and marketing environment around food” *(I12).

*“Companies will be working with you on the one hand but then lobbying against you on the other. Right? So these companies, especially the big ones... can be working with their sustainability team and the corporate social responsibility team on an initiative. And then you turn around and their government relations team in Washington DC is up on the Hill trying to kill that piece of legislation that would actually help solve the problem that you’re trying to work on with your sustainability team” *(I16).

The issue of whether to engage the private sector divides the nutrition community.^82^ Some argue that partnering with the private sector is impermissible because of the inherent conflicts of interest between corporations that profit from unhealthy food and public health collaborations. Others are willing to collaborate, finding it unrealistic to avoid the private sector because they are significant actors in food systems. Still others are open to communication but not to official partnership and instead engage at most in dialogue. Respondents expressed that these differences within the nutrition community affect the possibility of establishing PPPs:

*“[We have] an inability to come together on a coherent set of demands…as a function of some people have stronger ideological opposition to the industry and PPPs than others” *(I12).

*“You’ll always have vocal elements of the nutrition sector that come with a certain ideology…you’ll always have a group of people that will see nutrition as a public good that should be delivered by public channels…lots of the rhetoric around the[PPP] pushback relates to protecting populations from an evil private sector” *(I10).

###  Factors Hampering the Success of Nutrition-Related PPPs

These difficulties — nutrition’s complex causal pathways, weak global governance of nutrition, private sector power and transgressions, and nutrition community mistrust of private sector actors — present obstacles not only to the *establishment* of PPPs but to their *effectiveness* once created. Nutrition-related PPPs commonly face three problems that arise from effectiveness complexities: goal misalignment, power imbalances pertaining to resource contributions, and inadequate accountability mechanisms.

####  Goal Ambiguity and Misalignment

 The public sector comes to the table with public health objectives and the private sector with profit-making objectives. These goals often clash, hampering PPP performance (I2, I4, I6, I7, I12, I14, I15, I18, I19). Speaking about PPP goal ambiguity or lack of clarity, informants remark:

*“Depending on which nutrition scientists are in the room, in the case of the governance mechanisms, it’s not even clear that you can get agreement among the nutritionists” *(I12).

*“I find myself wanting there to be a firm contract and set of agreements in hand that are measurable and enforceable. Only then could both sides proceed or especially only then would the nutrition community or members of it proceed. Absent that kind of formal agreement, I don’t think that this mistrust issue can be overcome” *(I12).

Informants note that conflicts of interest underpin goal misalignment problems, a difficulty particularly acute in the nutrition research community where industries have funded research that benefits their own products and bottom-line (I2, I6, I13, I14, I16).^57^ One respondent compared this conflict of interest to the health sector’s relationship with pharmaceutical companies:

*“In nutrition, it feels like we’re still at a stage when everybody is kind of afraid of, especially from the public side, afraid of conflicts of interest rather than saying that’s the way it is. And we’re just going to be very pragmatic in how we approach that because food is coming almost entirely from the private sector and there is no way around it. Comparing the health sector to the nutrition sector, there is quite a gap in the way public sector interacts with businesses” *(I3).

 Others noted the dearth of individuals who might help transcend the mistrust between public and private sector actors. They note that the shared space between sectors and how to engage within it is unclear (I10, I18):

*“There aren’t that many people out there practiced at bridging these sectors. They usually come from their own sector, and they reach out into the other one, and they pretend they can talk the talk. But there’s very few examples of people that actually understand and empathize with the motivations of the various sectors and can serve as a bridging agent” *(I10).

####  Resource Imbalances

Resource problems also hamper PPP performance.^78^ The PPP mechanism is partly designed to address the issue of affordability by pooling resources from various sources to overcome the limited funding available in the public sector, which often brings in-kind contributions to the table. Governments do not invest enough in nutrition within their national budgets nor in official development assistance (ODA) with less than 1% of ODA going towards nutrition.^112^ However, with any partnership there is a tendency to value tangible financial resources above intangible resources such as capacities, expertise, reputation or networks.^78^ Hence partners committing more financial resources — almost always the private sector — tend to have greater bargaining power. This unequal power relationship within PPPs can create an environment in which weaker partners feel detached from the decision-making and management processes.^78^

Another resource problem is that a large number of informal or small-scale players in the private sector lack capacity to effectively engage in the partnership and deliver on the agreed-upon goals.^15,113^ They do not have the technical know-how or human capacity to effectively engage in the partnership and deliver on the agreed-upon goals (I1, I9. I10, I14, I18).^15,114,115^ While SMEs are flexible and can quickly respond to consumer demands, their ability to address growing concerns of food safety, traceability and health and environmental sourcing constrains their ability to partner effectively.^116-118^ One interviewee expressed concern for SEMEs:

*“SMEs-- I think they are willing but haven’t necessarily the capacity or the know-how, how to work it” *(I18).

Nevertheless, fortification and supplementation programs have multiple examples of where SMEs have successfully developed partnerships with the public sector.^38^

####  Weak Accountability

Nutrition-related PPPs also face problems with accountability and transparency (I4, I6, I8, I9, I12, I15).^12,30,63,104,119,120^ Accountability means:

1. Answerability: key actors provide an account of their decision and actions to relevant stakeholders using a trusted, transparent, responsive, credible and inclusive process that provides meaningful and verifiable information^104^; and
2. Enforceability: key actors comply with established standards and codes of conduct, and are subject to penalties or restrictions when they do not deliver on their pledges, commitments and obligations.^119^

A lack of civil society engagement hampers nutrition PPP accountability.^121^ However civil society organizations are key to holding governments and their partners to account. Vehicles such as the Civil Society Mechanism of the UN Committee on World Food Security could be more empowered to play a more significant role in ensuring that PPPs formed meet certain transparency and ethical metrics. As one respondent expressed:

*“There is a lack of understanding about how we engage civil society who can add some accountability perspective, and sometimes evidence in terms of how not just which policies should be designed but downstream through implementation, the actual impacts of those policies, particularly to vulnerable groups” *(I15).

Weak monitoring and evaluation mechanisms also make accountability difficult (I1, I5, I6, I12). Nutrition-related PPPs have rarely been subject to independent evaluation, a function in part of the fact that most are new.^79^ The earlier mentioned complexity of detecting causality also poses a problem for evaluation. As one respondent puts it:

*“The resources required for a great evaluation or the resources required for proper monitoring and interpretation and enforcement could be a stumbling block, likely because the complexity of the problem is such that it would take enormous resources to untangle what’s actually going on and where the accountability or blame lies if the articulated goals are not being achieved” *(I12).

 In nutrition, there are examples of effective accountability mechanisms. For instance, the Access to Nutrition Index ranks the world’s largest food and beverage companies on their nutrition-related commitments, practices and performance globally. This can provide a starting point for public sector entities to assess these companies’ suitability to partner based on their nutrition policies, product profiles and marketing strategies.

There are learnings from other sectors as well. The health sector provides insight for creating successful accountability mechanisms and partnership arrangements including the GAVI Vaccine Alliance and the non-profit organization PATH, which have established successful PPPs in public health.^122^ There are also many examples of where agriculture PPPs have worked effectively. ^123,124^ For example, one PPP examined from the literature established that improved women’s access to training, marketing, extension and financial services in producing local vegetables in India.^125^

## Discussion

 In the field of nutrition, discussion on the legitimacy and effectiveness of PPPs is polarized. Some argue that PPPs offer the potential to improve diet and nutrition outcomes by harnessing resources, reach, relationships and knowledge from both government and private sector actors. Others express deep mistrust of the private sector, viewing the food industry as prioritizing profit to the detriment of public health and as a cause of malnutrition, and therefore incapable of being part of the solution.

Of the seven factors that hinder success highlighted in this study, the issues of trust and power imbalances are the most challenging to overcome. Trust is particularly difficult to generate when many food and beverage industry players market products that harm public health and the environment. Lessons from other sectors reveal that effective partnerships depend on trust, transparent information sharing and effective management of conflicts of interest.^33,124,126-129^ Private sector actors also mistrust those in the public sector, shaping the former’s interest in partnership.^119^

With power imbalances, more established private-sector actors, particularly those that have undergone consolidation and have significant shares of key markets, have been known to use their power to override government voice and agency to set agendas.^43,93-97^ Unequal power relationship within PPPs can create an environment in which weaker partners (including local public and even SMEs, civil society organizations, and community organizations) feel detached from the decision-making and management processes of partnerships.^78^ This detachment can lead to reduced ownership and agency of these actors, which can threaten dialogue on accountability and transparency. Some governments find regulating the nutrition and food space challenging due to a lack of capacity to govern, or the “will to govern” the multiple actors involved, amongst which the private sector tends to dominate.^127^

 There are few third-party evaluations of PPPs, and those reports that exist provide limited guidance on how to construct PPPs that serve nutrition outcomes. Much of the literature focuses on the power of big business in the food sector, particularly with regard to their ability to influence public sector nutrition research specifically. There is much less published on how PPPs operate in practice for diet and nutrition outcomes—how these partnerships are formulated and structured, and how conflicts of interest are prevented, minimized and managed.

 The dearth of consideration of independent third-party evaluations of PPPs—because few exist—is a limitation of this study. Another limitation is that some experts who had strong aversion towards PPPs did not want to be interviewed because their view was that PPPs should not exist, and therefore, an evaluation was pointless. These limitations notwithstanding, this study was comprehensive in drawing on interviews with a wide range of experts from both public and private sectors, and an extensive literature in nutrition related to private sector engagement and controversies in nutrition research.

## Conclusion

 With the renewed attention on food systems, in particular the UN Food Systems Summit in 2021, there is an interest in how to effectively engage the private sector in food systems that would result in positive outcomes for public health nutrition, environmental sustainability and equity. The call for PPPs will be on the table at the Summit for consideration in which accountability mechanisms and declarations of interest will need to be clearly stated and established.

If PPPs are considered as a mechanism to address malnutrition and diets, the terms of partnership or more broadly, engagement, should be led by government, and the private sector should be steered to understand government priorities to promote healthy diets and nutrition. Many PPPs are currently impeded by constraints such as: lack of the prerequisite technical skills, limited resources, power imbalances, and lack of trust. In order to address trust deficits, it would be necessary for outside brokers to bring public and private sectors actors together. Trust issues cannot be resolved without direct involvement of the actors affected. Transparency and accountability are crucial for PPPs to work effectively and those accountability structures should track progress and sanction poor progress or inappropriate behavior made by partners.^128^ Additionally, there is a need to strengthen the evidence base in order to share and build upon lessons from the successes, as well as the challenges, of creating sustainable PPPs.

It is important to consider whether a partnership is necessary for tackling a specific public health nutrition objective. Hawkes and Buse^130^ suggest that before engaging with private sector, three questions should be answered:

1. Would engaging with private sector help achieve the objective faster and more effectively?
2. Would the interests involved (on both sides) enhance or threaten the likelihood of achieving the specific objective as well as longer-term public health objectives?
3. If interaction is a viable option, what form of engagement would most effectively achieve the objective while accounting for the different interests: a real partnership or less formal type of collaboration?

The issues that are most amenable to partnerships are those with clear causal pathways to improved nutrition, and ones where private sector interests are aligned with rather than in opposition to improved public health.

 Strengthened accountability systems would support government leadership and stewardship, incentivize private sector actors to include nutrition among its goals, and reinforce the engagement of civil society in creating demand for healthy food environments and monitoring progress towards the nutrition agenda objectives. But processes of engagement need to be transparent, open and inclusive. Engagement should not compromise any individual organization’s independence or reputation, and mutual accountability towards public nutrition goals should be the main goal. Governments must be the enactors, through mandatory regulation, to manage conflicts of interest. Voluntary or self-regulation of the private sector by the private sector is a less viable option to institute meaningful change and trust.

Governments need to exert power and shepherd their food systems in the directions that promote public health. They must create meaningful food-based dietary guidelines and public procurement programs,^104^ as well as fiscal instruments such as taxes on soda and unhealthy junk food, and regulate advertising junk food to children^103^ to keep the private sector in check. Only when these factors are in place and better public health nutrition outcomes are assured through them can PPPs be considered.

## Acknowledgements

 The authors would like to thank those who took the time to interview and review earlier versions of the manuscript. We are not including their names for anonymity sake.

## Ethical issues

 The study protocol was cleared through the Institutional Review Board of the Johns Hopkins University, which granted the study exempt status because it focused on public policy and was deemed to pose minimal risk to informants.

## Competing interests

 Authors declare that they have no competing interests.

## Authors’ contributions

 JF, YRS and JS contributed to the design and overall conception of the research. YRS, JF, TS and SD performed the implementation of the literature research and conducted interviews. YRS, TS and SD analyzed the data and performed the formal analysis. JF, YRS, JS and TS contributed to the writing and the final version of the manuscript.

## Funding

 Funding was received from the Global Alliance for Improved Nutrition to carry out this work.

## Authors’ affiliations

^1^The Nitze School of Advanced International Studies, Johns Hopkins University, Baltimore, MD, USA. ^2^The Bloomberg School of Public Health, Johns Hopkins University, Baltimore, MD, USA. ^3^The Berman Institute of Bioethics, Johns Hopkins University, Baltimore, MD, USA. ^4^Independent Consultant, Singapore, Singapore. ^5^Independent Consultant, New Delhi, India.
